# Enhancing the Performance of Perovskite Solar Cells by Introducing 4-(Trifluoromethyl)-1*H*-imidazole Passivation Agents

**DOI:** 10.3390/molecules28134976

**Published:** 2023-06-24

**Authors:** Wei Hua, Qiaoli Niu, Ling Zhang, Baoxiang Chai, Jun Yang, Wenjin Zeng, Ruidong Xia, Yonggang Min

**Affiliations:** 1 State Key Laboratory of Organic Electronics and Information Displays & Institute of Advanced Materials (IAM), Nanjing University of Posts & Telecommunications, Nanjing 210023, China; 2 The School of Materials and Energy, Guangdong University of Technology, Guangzhou 510006, China

**Keywords:** perovskite solar cells, passivation, additives, THI

## Abstract

Defects in perovskite films are one of the main factors that affect the efficiency and stability of halide perovskite solar cells (PSCs). Uncoordinated ions (such as Pb^2+^, I^−^) act as trap states, causing the undesirable non-radiative recombination of photogenerated carriers. The formation of Lewis acid–base adducts in perovskite directly involves the crystallization process, which can effectively passivate defects. In this work, 4-(trifluoromethyl)-1H-imidazole (THI) was introduced into the perovskite precursor solution as a passivation agent. THI is a typical amphoteric compound that exhibits a strong Lewis base property due to its lone pair electrons. It coordinates with Lewis acid Pb^2+^, leading to the reduction in defect density and increase in crystallinity of perovskite films. Finally, the power conversion efficiency (PCE) of PSC increased from 16.49% to 18.97% due to the simultaneous enhancement of open-circuit voltage (V_OC_), short circuit current density (J_SC_) and fill factor (FF). After 30 days of storage, the PCE of the 0.16 THI PSC was maintained at 61.9% of its initial value, which was 44.3% for the control device. The working mechanism of THI was investigated. This work provides an attractive alternative method to passivate the defects in perovskite.

## 1. Introduction

In recent years, halide perovskite solar cells (PSCs) have developed rapidly, and their performance has achieved significant improvements. Their power conversion efficiency (PCE) has increased at an astonishing rate from the initial 3.8% to over 25% [1,2], which is close to that of the currently commercialized silicon-based solar cells, demonstrating the enormous commercialization prospects in the field of photovoltaics. The excellent performance of PSC is mainly due to the unique properties of halide perovskite, such as its high absorption coefficient, low binding energy, long carrier diffusion length and high carrier mobility. Meanwhile, halide perovskite thin films can be deposited through the low-temperature solution processing technology, exhibiting its great potential for flexible devices [3,4].

However, at present, PSCs face a serious challenge, which that the deep-energy-level defects in perovskite films severely limit their PCE and stability. The film quality of perovskite plays a vital role in achieving highly efficient PSCs. Despite great advances in high-quality perovskite, the inherently soft lattices of polycrystalline films deposited by the solution-processing technology present weak binding character, resulting in rich defects [5,6]. Defects were generated during the in situ crystallization of perovskite film, of which the deep-energy-level defects caused non-radiative recombination losses during device operation, leading to the performance degradation. Moreover, these defects can bring about ion migration under illumination or electric field, which reduces the stability of perovskite [7,8,9]. Therefore, it is necessary to eliminate the deep-energy-level defects in perovskite films to improve the performance of PSCs. Deep-energy-level defects in MAPbI_3_ thin films are mainly point defects, including uncoordinated Pb^2+^, uncoordinated halide ions, Pb clusters and Pb-I antisite defects [10,11]. Thus, forming chemical bonds is a promising way to eliminate uncoordinated Pb^2+^ or halide ions [12]. According to the soft material nature of perovskite, weak interactions such as coordination bonds, ion bonds, and hydrogen bonds are used, among which, coordination bonds based on Lewis acid–base theory are very promising [11]. Imidazole is a typical amphoteric compound, in which the nitrogen has a lone pair of electrons exhibiting strong basicity and weak acidity due to the combination of an acid proton with another nitrogen molecule. Thus, the imidazole group is likely to coordinate with Lewis acid and Lewis base defects, forming Lewis acid–base adducts. For example, imidazole was doped into the perovskite absorption layer to realize the reduction in defect density [13]; 4-iodo-1H-imidazole was introduced into the perovskite precursor solution to prevent defect formation, leading to a significant elevation in the open-circuit voltage (V_OC_) of PSC [14]; imidazolium iodide (ImI) applied on the surface of MAPbI_3_ passivated the iodide vacancies of the perovskite by occupying the vacant sides in the crystal lattice, which minimized recombination losses [15]; 4-imidazoleacetic acid hydrochloride (ImAcHCl) was employed to modify the surface of electron transport layer SnO_2_ [16], leading to the passivation of defects via interaction between imidazolium cation in ImAcHCl and iodide anion in perovskite; the surface modification of TiO_2_ was achieved using 1-butyl-3-methylimidazolium tetrafluoroborate, resulting in increased PCE and eliminated hysteresis in planar PSCs [17]; the incorporation of imidazole bromide functionalized graphene quantum dots (I-GQDs) on the surface of the electron transport layer not only reduced the interface defects but also improved the film quality of FAPbI_3_ perovskite [18].

In this study, imidazole derivative 4-(trifluoromethyl)-1H-imidazole (THI) was introduced into the perovskite precursor solution to passivate defects in the perovskite films. Due to its voluminous size, THI will not enter into the perovskite lattice but can act as an ionic. It was shown that THI-added perovskite films have better crystallinities and larger grain sizes, which effectively reduces the density of defect states and eliminates non-radiative recombination. The doping concentration of THI in the perovskite precursor solution was optimized, which was 0.16 wt%. The PCE of PSC was significantly enhanced from 16.49% of the control device to 18.97%. The work mechanism of THI was thoroughly investigated by using X-ray photoelectron spectroscopy (XPS), a scanning electron microscope (SEM) and an electrochemical impedance spectrum (EIS). This work provides an alternative passivate agent to passivate defects of perovskite film.

## 2. Results and Discussion

**Figure 1:**
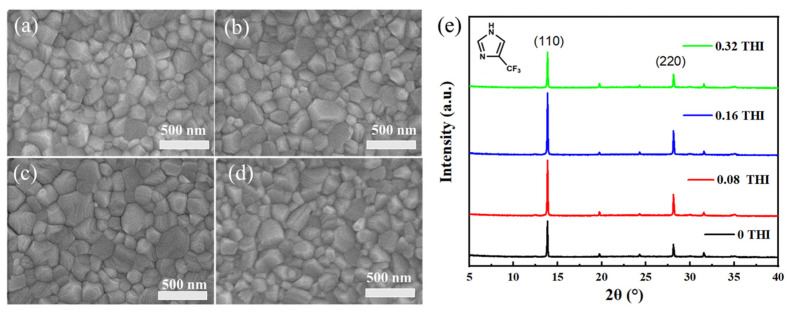
Top-view SEM images of perovskite films: (**a**) 0 THI, (**b**) 0.08 THI, (**c**) 0.16 THI and (**d**) 0.32 THI; (**e**) XRD patterns of perovskite films based on different concentration of THI; inside is the molecular structure of THI.

The effect of THI addition on the morphology of perovskite thin films was investigated, the SEM images of which are shown in Figure 1. The perovskite films prepared from different weight ratios of THI in the perovskite precursor solution (0 wt%, 0.08 wt%, 0.16 wt% and 0.32 wt%) were named 0 THI, 0.08 THI, 0.16 THI and 0.32 THI, respectively. It could be observed that all the perovskite films were dense and pinhole-free with full coverage on the substrate. But the grain sizes varied greatly, which were estimated using Nano Measure 1.2 software and are summarized in Table 1. It can be seen that the grain size of MAPbI_3_ thin films increased first and then decreased with the increasing doping ratio of THI from 0 wt%, 0.08 wt% and 0.16 wt% to 0.32 wt%. The mean grain size values of all THI-doped perovskite films were larger than that of the pristine film. The 0.16 THI perovskite film had the largest average grain size (223.78 nm), while that of the 0 THI perovskite films was 177.44 nm. The significantly increased grain size indicates that THI doping enhanced the crystallinity of the perovskite films.

X-ray diffraction (XRD) patterns were also collected to further study the crystal structure and crystallinity of perovskite films (Figure 1e); inside was the molecular structure of THI, which is also shown in Figure S1. All the XRD patterns show two significant peaks at 13.90° and 28.17°, corresponding to the (110) and (220) crystal planes of MAPbI_3_, respectively. This indicates that all MAPbI_3_ films had an orthorhombic crystal structure, and the addition of THI did not change the component of perovskite. Meanwhile, the intensity of the XRD peaks changed significantly after doping THI. In terms of the peak at 13.90°, the intensities of 0 THI, 0.08 THI, 0.16 THI and 0.32 THI perovskite films were 4605, 6940, 7831 and 4669, respectively. In addition, it was also found that the full width at half maximum (FWHM) of (110) diffraction peak (Table 1) was narrowed as a result of the THI doping. The uplifted peak intensity and narrowing of FWHM of XRD patterns indicate that THI doping enhanced the crystallinity of MAPbI_3_ films. The parabolic-shaped changing trend of peak intensity with the concentration increase in THI shows that 0.16 THI film had the strongest diffraction peak intensity. Thus, 0.16 wt% is the optimum doping concentration of THI in consideration of the crystallinity of the perovskite films. This result is in accordance with the SEM images of perovskite films.

**Figure 2:**
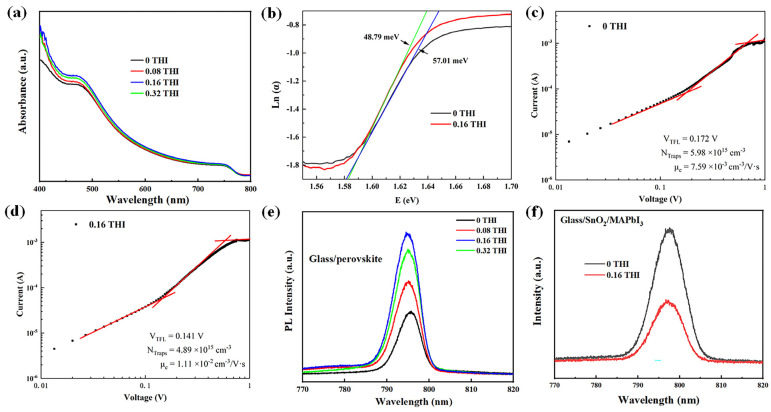
(**a**) UV-Vis spectra of perovskite films based on different concentrations of THI in the perovskite precursor solution; (**b**) Urbach energy plot; J–V characterizations of electron-only devices with structure of ITO/SnO_2_ (20 nm) /MAPbI_3_ (260 nm)/PCBM (70 nm)/Ag (100 nm); (**c**) control device and (**d**) 0.16 THI device; (**e**) PL spectra of perovskite films on glass; (**f**) PL spectra of perovskite films on SnO_2_/glass.

The changes in the morphology of perovskite thin films are often accompanied by variations in their light absorbance. The ultraviolet–visible (UV-Vis) absorption spectra of perovskite films were measured, as shown in Figure 2a. The absorption spectra of MAPbI_3_ films with different THI doping concentrations all showed the same shapes with two distinct absorption peaks at 480 nm and 740 nm, indicating the unchanged crystal structure of MAPbI_3_ films, which is consistent with the XRD patterns. After doping THI, the absorbance of perovskite film was enhanced in the range of 400–700 nm, which can be attributed to the improved crystallinity of MAPbI_3_ films [19,20]. 

The enlarged grain size of perovskite film means reduced defect density. Thus, Urbach energy (*E_u_*) was calculated by fitting the exponential part of the absorption edge, according to Equation (1). The Urbach tail can reflect the defect density of perovskite [21], the change in which is consistent with the change in defect density. The greater the Urbach energy, the higher the defect density [22].

(1) $$\alpha \left(E\right)=\alpha_{0}\times exp\left[\frac{E-E_{0}}{E_{u}}\right]$$

where α is the absorption coefficient and *E* is the photon energy. *E_u_* value can be determined from the inverse of the slope of the linear fit of *Ln*(*α*) against *E*. The calculated *E_u_* values are 57.01 meV and 48.79 meV for the pristine and 0.16 THI films, respectively. The decrease in the *E_u_* value indicates the decrease in the defect density in the perovskite films after doping with THI [23].

The space-charge-limited current technique is often applied to electron- or hole-only devices to estimate the trap densities in semiconductors. Figure 2c,d show the dark I-V curves of electron-only devices in double logarithmic coordinates based on the pristine and 0.16 THI perovskite films. The structure of an electron-only device consists of indium tin oxide (ITO)/SnO_2_ (40 nm)/MAPbI_3_ (260 nm)/[6,6]-phenyl C61 butyric acid methyl ester (PCBM) (70 nm)/Ag (100 nm). The linear region at low bias voltage corresponds to the ohmic response. A trap-filling process is identified by the significant increase in the current injection in the intermediate region [24]. At high voltage, it is the space-charge-limited current (SCLC) region. The trap-filled-limited voltage (*V_TFL_*) refers to the kink point of the ohmic and TFL regions [25,26]. Typically, the density of defects ($N_{defects}$) can be calculated using Equation (2) [27]:

(2) $$N_{defects}=\frac{2\varepsilon \varepsilon_{0}V_{TFL}}{eL^{2}}$$

where ε is the relative dielectric constant of MAPbI_3_ (*ε* = 32) [27], $\varepsilon_{0}$ is the vacuum permittivity, *L* is the thickness of the perovskite film, *e* is the unit charge, and *V_TFL_* is the TFL voltage. It can be read from Figure 2c,d that the *V_TFL_* values of the control device and the 0.16 THI device are 0.172 V and 0.141 V, respectively. Accordingly, the calculated trap densities of the control device and the 0.16 THI device were 5.98 × 10^15^ cm^−3^ and 4.89 × 10^15^ cm^−3^, respectively. This further confirmed the diminishment of trap densities in perovskite film after the addition of THI. 

The decrease in defect density is conducive to the transportation of charge carriers, which was verified by estimating the carrier mobility via Mottley–Gurney’s law (3) from the SCLC region [28]:

(3) $$J_{D}=\frac{9\varepsilon \varepsilon_{0}\mu V_{b}^{2}}{8L^{3}}$$

wherein *V_b_* is the applied voltage; *J_D_* is the measured current density; *μ* is the mobility of charge carriers; *L* is the thickness of the perovskite light-absorbing layer; *ε* is the relative dielectric constant of MAPbI_3_ (*ε* = 32). The calculated electron mobility (*μ_e_*) of the 0.16 THI device was 1.11 × 10^−2^ cm^2^ V^−1^ s^−1^, which was much higher than the corresponding value of the control device: 7.59 × 10^−3^ cm^2^ V^−1^ s^−1^.

The steady-state photoluminescence (PL) spectra of perovskite films on glass were usually used to inspect the non-radiative recombination, which was shown in Figure 2e. The PL intensity of all THI-doped perovskite films was higher than that of the pristine one, implying the enhanced radiative recombination. Thus, the non-radiative recombination was reduced, which was mainly supported by the deep-level traps [29]. The 0.16 THI perovskite film had the highest PL intensity, suggesting the best film quality with the minimum trap density. This was in accordance with the SEM and XRD results. In addition, there was a small blue shift in the PL peak position after the addition of THI relative to the pristine film, which was also indicative of a reduction in defect density in the perovskite films [30]. Thus, the addition of THI passivated the defects in the MAPbI_3_ films. Moreover, PL is an effective method for studying the carrier dynamics at the interface. Effective carrier separation will cause a reduction in carrier radiative recombination. Thus, the lower PL intensity of the perovskite films on the charge transport layer indicates less carrier recombination, suggesting more effective carrier extraction. The PL spectra of the ITO/SnO_2_/MAPbI_3_ films were measured, where SnO_2_ was the electron transport layer, as shown in Figure 2f. The PL intensity of the 0.16 THI MAPbI_3_ film was lower than that of the pristine film, indicating the enhanced electron extraction after THI doping.

**Figure 3:**
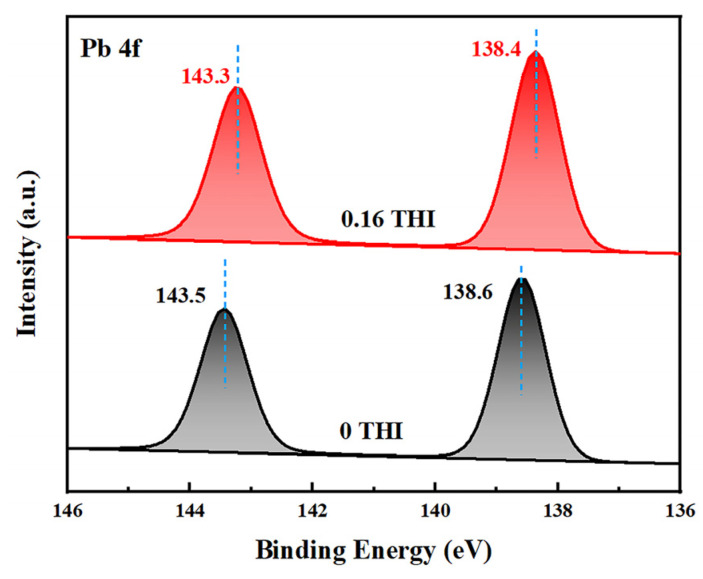
XPS spectra of Pb 4f of the pristine and 0.16 THI perovskite films.

The above experimental results demonstrate that a THI additive in the precursor solution passivates the defects in perovskite films, resulting in increased light absorbance, enhanced PL intensity and more efficient electron extraction. To investigate the passivation mechanism of THI, X-ray photoelectron spectroscopy (XPS) was used to study the interaction between THI and MAPbI_3_. Figure 3 shows the XPS spectra of Pb 4f of the pristine and 0.16 THI MAPbI_3_ films. It shows two characteristic peaks corresponding to the spin–orbit splitting of the Pb 4f7/2 and Pb 4f5/2 components, respectively [31,32]. After doping 0.16 wt% THI, both the two peaks shifted downward, from 143.5 eV and 138.6 eV of the pristine film to 143.3 eV and 138.4 eV, respectively. The lower binding energy suggested the interaction between the imidazole group in THI and the uncoordinated Pb^2+^ in MAPbI_3_ [33]. In addition, the XPS spectra of I 3d of the pristine and 0.16 THI MAPbI_3_ films were also collected, as shown in Figure S1. The characteristic peaks of I 3d of the MAPbI_3_ film were 619.3 and 630.8 eV [32], which did not change after the addition of THI. This implies that there is no interaction between THI and I^−^, or the interaction is too weak to be detected. It can be ascribed to the weak Lewis acid property of THI.

**Figure 4:**
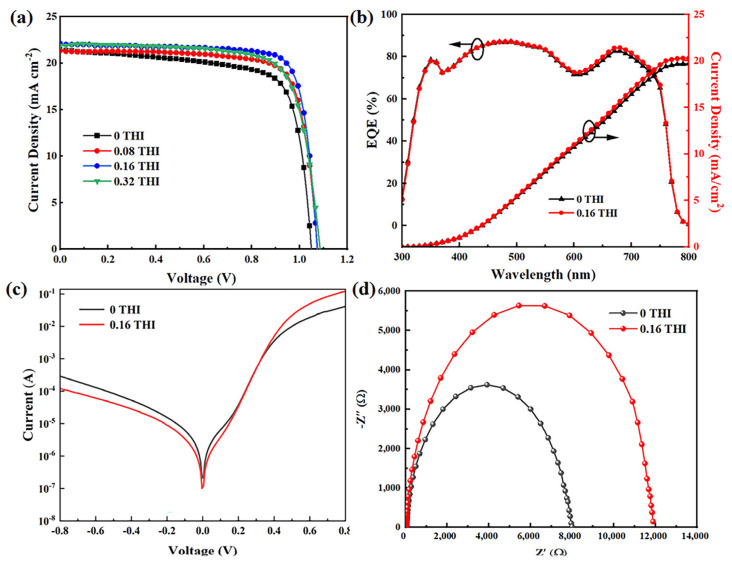
(**a**) J−V curves of PSCs based on different THI doping concentrations; (**b**) IPCE curves of PSCs, (**c**) dark I-V curves and (**d**) Nyquist plots of EIS of the control and 0.16 THI devices.

The effects of THI additives on the performance of PSCs were characterized by fabricating devices with structures of ITO/NiOx (20 nm)/MAPbI_3_ (260 nm)/PCBM (70 nm)/2,9-dimethyl-4,7-diphenyl-1,10-phenanthroline (BCP) (5 nm)/Ag (100 nm), which is illustrated in Figure S2. The current density–voltage (J–V) curves of PSCs based on perovskite films with different concentrations of THI are shown in Figure 4a, and the corresponding performance parameters are summarized in Table 2. It can be seen that the V_OC_, short circuit current density (J_SC_) and fill factor (FF) of PSCs were all increased after the addition of THI. With the concentration increase in THI, the performance of PSC increased first and then decreased. When the doping concentration of THI was 0.16 wt%, the device performance reached the maximum. The V_OC_, J_SC_ and FF of the control device were 1.05 V, 21.39 mA cm^−2^ and 73.44%, respectively, which were 1.07 V, 22.04 mA cm^−2^ and 80.19% for the 0.16 THI-based PSC, resulting in an enhancement in PCE from 16.49% to 18.97%. The PCE statistics of PSCs are shown in Figure S3, which show narrow distributions for both the control and 0.16 THI devices. Shockley Read Hall (SRH) recombination is considered to be the dominant pathway leading to the V_OC_ loss in PSC, which is mainly caused by deep-level defects in perovskite films [11]. Thus, the passivation of defects in perovskite films by adding THI led to the increase in the V_OC_ of PSC. Incident photon-to-current efficiency (IPCE) data were collected to verify the increase in the J_SC_ of PSCs, as shown in Figure 4b. The 0.16 THI PSC exhibited a stronger spectral response in the whole visible wavelength range compared with the control device. The integrated current density was 19.71 mA cm^−2^ and 20.30 mA cm^−2^ for the control and 0.16 THI device, respectively. The reduction in defects in perovskite films is beneficial for the transportation of charge carriers, which is the major factor that caused the improvement in the J_SC_ of PSC.

To investigate the charge carrier transport dynamic, the dark I-V and Nyquist plot of EIS of the control and 0.16 THI PSCs were collected. As shown in Figure 4c, at a negative voltage, the lower current of the 0.16 THI device compared with the control device suggested a smaller leakage current, which was in an inverse relationship with the shunt resistance (R_sh_). At a high positive voltage, the current was in an inverse relationship with the series resistance (R_s_). The larger current of the 0.16 THI device implied a smaller R_s_, which resulted from the improved perovskite film with less defects [34]. Both the larger R_sh_ and smaller R_s_ corresponded to a higher FF [35]. Thus, the FF of PSC increased with the addition of THI.

In addition, EIS is a powerful tool to investigate properties of materials and electrode reactions. The Nyquist plots of EIS were depicted to obtain the composite resistance (R_rec_) of PSCs, as shown in Figure 4d. R_rec_ corresponds to the low-frequency region of the Nyquist plots, which can be quantitatively evaluated. The value of the control device (6443 Ω) was much lower than that of the 0.16-THI-based device (17,637 Ω), indicating the suppressed charge carrier recombination after THI doping. Thus, it contributed to the collection of electrons and holes, and therefore the elevation in J_SC_ [36].

**Figure 5:**
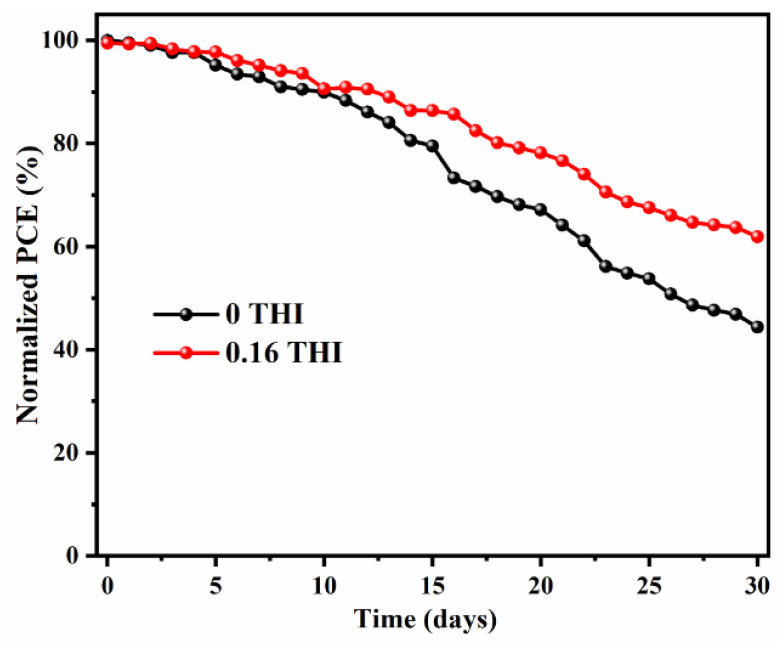
Thirty-day storage stability of PSCs without encapsulation by storing in N_2_-filled glove box.

In addition to improving efficiency, device stability is also critical in achieving commercialization. The 30-day storage stability of PSCs without encapsulation was checked by storing them in a N_2_-filled glove box (with the content of both water and O_2_ being less than 1 ppm) and tested daily. The normalized PCEs as a function of time were recorded, as shown in Figure 5. The J−V curves of the PSCs after being stored for 1 day and 30 days are shown in Figure S4. After 30 days of storage, the PCE of the 0.16-THI-added device was maintained at 61.9% of its initial value, while it dropped to 44.3% for the control device. Obviously, the device based on THI-doped perovskite was more stable than the control device.

## 3. Discussion

XPS spectra demonstrated the interaction between THI and Pb^2+^, from which, it can be speculated that Lewis base–acid adducts were formed because of the strong Lewis base property of THI and Lewis acid property of Pb^2+^. The formation of Lewis base–acid adducts may slow down the perovskite crystal growth, beneficial to the formation of large grains [13]. The passivation of defects was also confirmed by the calculated defect density values from J−V of the electron-only device and E_U_ values. The lowered defect density in perovskite films elevated the carrier mobility, which was inspected using Mottley–Gurney’s law from the J−V of electron-only device. 

The improved film quality of perovskite with increased crystallinity, reduced defect density and elevated carrier mobility boosted its light absorbance and improved the extraction efficiency of electrons. Therefore, the V_OC_, J_SC_ and FF of PSCs were all improved, leading to the great enhancement in PCE.

## 4. Materials and Methods

### 4.1. Materials

ITO substrate, PbI_2_ (99.99%), Pb(Ac)_2_ (99.99%), MAI (99.5%) and PCBM (99.5%) were purchased from Xi’an Polymer Light Technology Corp, Xi’an, China. BCP (99%) was purchased from Borun New Material Technology Corp, Ningbo, China. NiOx nanoparticles were synthesized in our lab according to the reference [36]. NiCl_2_·6H_2_O was purchased from MACKLIN, Shanghai, China. DMF (99.8%), chlorobenzene and DMSO were purchased from Sigma Aldrich, St. Louis, MO, USA. THI was purchased from J&K Scientific, Beijing, China. Ag (99.99%) was purchased from China New Metal, Beijing, China. SnO_2_ (15% in H_2_O colloidal dispersion) was purchased from Alfa Aesar, Thermo Fisher Scientific, Heysham, UK. All materials were used as received without further purification.

By co-dissolving MAI, PbI_2_ and Pb(Ac)_2_ powder in DMF with a molar ratio of 2.2:0.4:0.6, the perovskite precursor solution with a concentration of 1 mol/L was prepared. The addition of THI was performed by doping it in the perovskite precursor solution with weight ratios of 0.08 wt%, 0.16 wt% and 0.32 wt%, which was stirred for at least 6 h in a nitrogen-filled glove box before use. The PCBM solution was prepared by dissolving it in chlorobenzene with a concentration of 20 mg/mL. NiOx was ultrasonically dispersed in deionized water at a concentration of 20 mg mL^−1^ and filtered with a 0.45 µL water-based filter before using. 

### 4.2. Device Fabrication

The device structure of PSC was composed of ITO/NiOx (20 nm)/MAPbI_3_ (260 nm)/PCBM (70 nm)/BCP (5 nm)/Ag (100 nm). Before use, ITO substrates were ultrasonically cleaned in sequence with detergent solution, deionized water, acetone and ethanol, taking 15 min for each step. After cleaning, the substrates were dried at 60 °C for 30 min in a bake oven. Then, O_3_ plasma surface treatment was applied for 4 min to remove the chemical residues on the surface of the ITO and facilitate the deposition of NiOx thin film. The NiOx solution was spin-coated on ITO at 2500 rpm for 30 s and then annealed at 130 °C for 10 min under atmospheric conditions. Following this, the ITO/NiOx samples were transferred to a nitrogen-filled glove box (with the content of both water and O_2_ being less than 1 ppm). The perovskite precursor solution was spin-coated at 4000 rpm for 30 s and then annealed on a hot plate at 100 °C for 15 min to form perovskite thin films. After that, the PCBM solution was spin-coated on top of the perovskite layer with a speed of 1200 rpm for 30 s and then thermal annealed on a hot plate at 100 °C for 2 min. Finally, a 5 nm thick BCP layer and a 100 nm thick Ag layer were sequentially thermally deposited under a pressure higher than 4 × 10^−4^ Pa. The active area defined by electrode masks was 0.09 cm^2^.

For the fabrication of electron-only devices with structures of ITO/SnO_2_/MAPbI_3_/ PCBM/Ag, SnO_2_ film was fabricated by spin-coating the SnO_2_ H_2_O colloidal dispersion on precleaned ITO at 3000 rpm for 30 s and then thermally annealing it at 150 °C for 30 min. Following this, MAPbI_3_, PCBM and Ag films were deposited using the above-mentioned processes.

### 4.3. Measurement and Characterization

XRD patterns of the perovskite films were obtained by using a Bruker D8 ADV ANCE X-ray diffractometer (Bruker Corp, Berlin, Germany) under the operation conditions of 40 kV and 40 mA. The absorption spectra were measured using a UV-visible spectrophotometer (UV3600, Shimadzu, Kyoto, Japan). The morphologies of perovskite films were observed via field emission scanning electron microscopy (FESEM, S4800 microscope, Hitachi Ltd., Tokyo, Japan). The steady-state PL was measured using an FLSP920 spectrometer (Edinburgh Instruments Ltd., Livingston, UK). X-ray photoelectron spectroscopy (XPS) was studied using a PHI Quantera SXM (ULVAC-PHI Inc., Tokyo, Japan). The current density–voltage (J−V) curves of the devices were measured using a Keithley 2400 Source Meter under an illumination of 1 sun (100 mW/cm^2^ AM 1.5 G, generated by a solar simulator Oriel Sol3A, Newport Corp., Irvine, CA, USA), which was calibrated with a standard Si photodiode. Incident photon-to-current efficiency (IPCE) data were collected by using a QTest Station1000 (Crowntch, Inc., Macungie, PA, USA). The electrochemical impedance spectrum (EIS) was tested using an electrochemical workstation (Zahner, Germany) under dark conditions.

Perovskite films for XRD, SEM, UV, PL and XPS tests were all prepared by spin-coating the perovskite precursor solution on ITO substrates with a concentration of 1 mol/L at 4000 rpm for 30 s. Then, the films were thermally annealed on a hot plate at 100 °C for 15 min. The films thicknesses were about 260 nm.

## 5. Conclusions

THI was introduced as an additive to the perovskite precursor solution. XPS spectra of Pb 4f in perovskite films verified the interaction between THI and uncoordinated Pb^2+^, which effectively reduced the defect density in perovskite film. Moreover, the crystallinity and grain size of perovskite films were significantly enhanced, leading to increased light absorbance, inhibited non-radiative recombination and enhanced charge carrier extraction. The doping concentration of THI was optimized in consideration of the performance of PSCs. With 0.16 wt% THI in the perovskite precursor solution, the PCE of PSCs increased from 16.49% to 18.97%. The storage stability of PSCs was also improved.

## Figures and Tables

**Table 1:** Grain size statistics, XRD peak intensity and FWHM values of diffraction peak of 13.90° for perovskite films with different doping ratios of THI.

Perovskite Film	Maximum (nm)	Minimum (nm)	Mean (nm)	Peak Intensity	FWHM (°)
0 THI	270.25	82.23	177.44	4605	0.0893
0.08 THI	377.68	109.47	180.09	6940	0.0881
0.16 THI	424.15	120.62	223.78	7831	0.0831
0.32 THI	293.58	131.93	199.54	4669	0.0884

**Table 2:** Summary of detailed performances parameters of PSCs.

PSCs	V_OC_ (V)	J_SC_ (mA cm^−2^)	FF (%)	PCE (%)
0 THI	1.05	21.39 ± 0.61	73.44 ± 2.12	16.49 ± 0.59
0.08 THI	1.07	21.26 ± 0.57	78.11 ± 1.97	17.87 ± 0.57
0.16 THI	1.07	22.04 ± 0.65	80.19 ± 1.83	18.97 ± 0.64
0.32 THI	1.08	21.99 ± 0.77	74.92 ± 2.04	17.91 ± 0.83

## Data Availability

Data sharing not applicable.

## Supplementary Materials

The following supporting information can be downloaded at: https://www.mdpi.com/article/10.3390/molecules28134976/s1, Figure S1: The molecule structure of THI; Figure S2: The device configuration of PSCs. The films thicknesses were also included; Figure S3: PCE statistic of PSCs based on pristine (0 THI) and 0.16 THI perovskite film, 20 devices for each kind of device; Figure S4: J−V curves of the PSCs after being stored for one day and 30 days: (a) 0.16 THI-PSC and (b) control device.
