# Supplementary material for: Enhancing the Performance of Perovskite Solar Cells by Introducing 4-(Trifluoromethyl)-1H-imidazole Passivation Agents
Source: Molecules. 2023 Jun 24;28(13):4976. doi: 10.3390/molecules28134976 (PMC10343423; doi:10.3390/molecules28134976)
Supplement: Supplementary file 1 [file molecules-28-04976-s001.zip › molecules-2443505-supplementary.pdf]

Supplementary Information

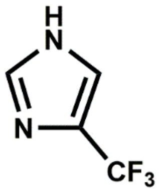

Figure S1. The molecule structure of THI.

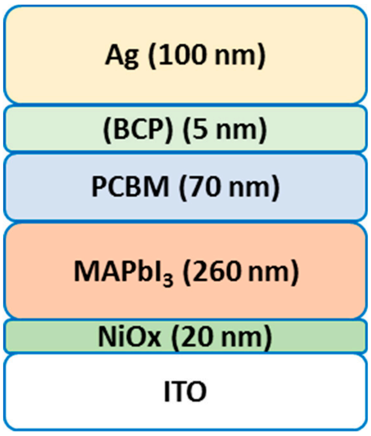

Figure S2. The device configuration of PSCs. The films thicknesses were also included.

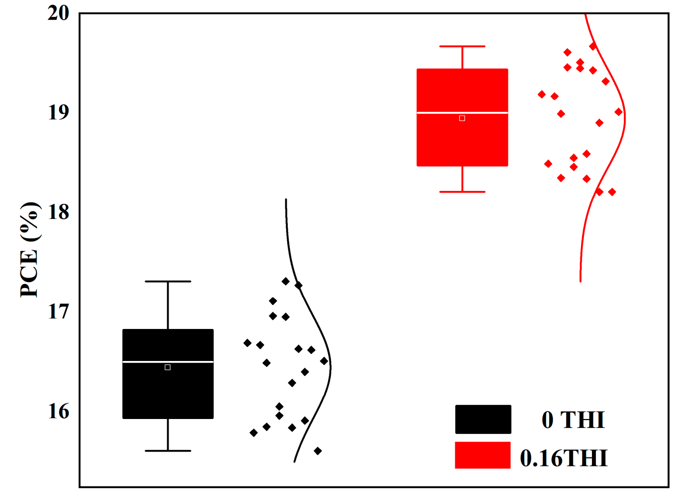

Figure S3. PCE statistic of PSCs based on pristine (0 THI) and 0.16 THI perovskite film, 20 devices for each kind of device.

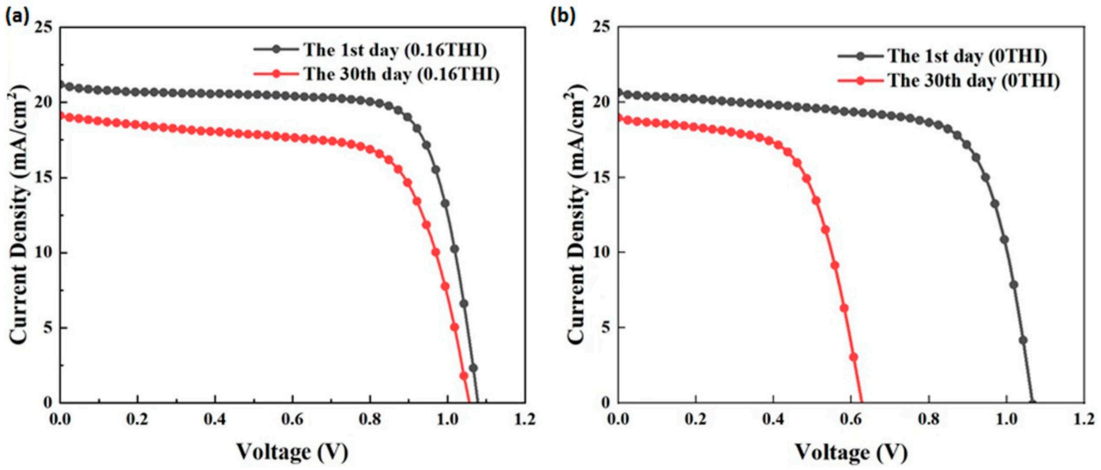

Figure S4. J-V curves of the PSCs after being stored for one day and 30 days: (a) 0.16THI-PSC and (b) control device.
